# Comparison of De Novo Assembly Strategies for Bacterial Genomes

**DOI:** 10.3390/ijms22147668

**Published:** 2021-07-17

**Authors:** Pengfei Zhang, Dike Jiang, Yin Wang, Xueping Yao, Yan Luo, Zexiao Yang

**Affiliations:** 1 Key Laboratory of Animal Diseases and Human Health of Sichuan Province, Sichuan Agricultural University, Chengdu 611130, China; 2018103006@stu.sicau.edu.cn (P.Z.); 2020103005@stu.sicau.edu.cn (D.J.); 13577@sicau.edu.cn (X.Y.); 41187@sicau.edu.cn (Y.L.); 13643@sicau.edu.cn (Z.Y.); 2 Collage of Veterinary Medicine, Agricultural University, Chengdu 611130, China

**Keywords:** long-read sequencing, genome assembly, protein prediction

## Abstract

(1) Background: Short-read sequencing allows for the rapid and accurate analysis of the whole bacterial genome but does not usually enable complete genome assembly. Long-read sequencing greatly assists with the resolution of complex bacterial genomes, particularly when combined with short-read Illumina data. However, it is not clear how different assembly strategies affect genomic accuracy, completeness, and protein prediction. (2) Methods: we compare different assembly strategies for *Haemophilus parasuis*, which causes Glässer’s disease, characterized by fibrinous polyserositis and arthritis, in swine by using Illumina sequencing and long reads from the sequencing platforms of either Oxford Nanopore Technologies (ONT) or SMRT Pacific Biosciences (PacBio). (3) Results: Assembly with either PacBio or ONT reads, followed by polishing with Illumina reads, facilitated high-quality genome reconstruction and was superior to the long-read-only assembly and hybrid-assembly strategies when evaluated in terms of accuracy and completeness. An equally excellent method was correction with Homopolish after the ONT-only assembly, which had the advantage of avoiding hybrid sequencing with Illumina. Furthermore, by aligning transcripts to assembled genomes and their predicted CDSs, the sequencing errors of the ONT assembly were mainly indels that were generated when homopolymer regions were sequenced, thus critically affecting protein prediction. Polishing can fill indels and correct mistakes. (4) Conclusions: The assembly of bacterial genomes can be directly achieved by using long-read sequencing techniques. To maximize assembly accuracy, it is essential to polish the assembly with homologous sequences of related genomes or sequencing data from short-read technology.

## 1. Introduction

Second-generation sequencing (SGS) platforms, such as Illumina, have significant limitations, although they are widely used in bacterial-genome research [1]. First, for a single laboratory, an SGS device requires significant capital investments (about USD 980,000), and the operation of the instrument has strict requirements in terms of the laboratory environment and the operators’ skills. Second, the process of SGS sample preparation and library construction is cumbersome. In addition, the sequencing process is time-consuming, and the output lags. Third, SGS platforms are based on PCR amplification for carrying out DNA molecule sequencing. Thus, they are limited by amplification and sequencing bias complications, and the read lengths are restricted to a few hundred bases. Short-read lengths make it difficult to achieve complete de novo assembly for genomes that include longer repetitive elements. The structures of repetitive regions include, for example, resistance gene cassettes, insertion sequences, and transposons.

The first established long-read technology was that of Pacific Biosciences (PacBio), which uses a sequencing-by-synthesis approach [2]. For the PacBio Sequel machine, the average length of reads is in the range of 10–14 kb. The error rate for raw reads is about 13–15%. The throughput can reach up to about 30 Gb per run. However, the cost of genome sequencing with the PacBio technology is still relatively high, as it involves a high initial cost for the platform (about USD 350,000) and about USD 100 per Gb.

**Figure 1:**
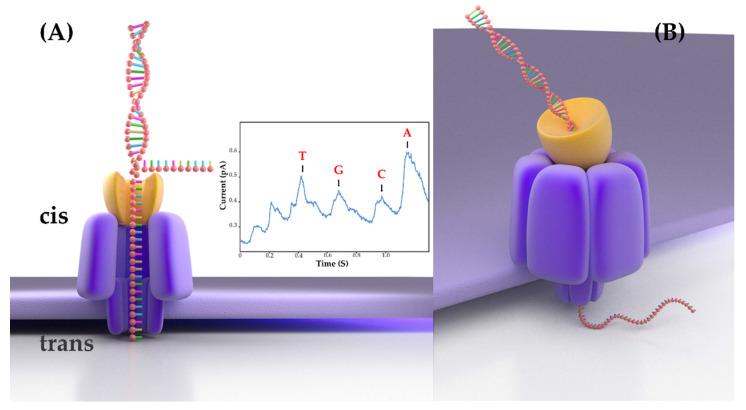
Nanopore sequencing. (**A**) Single-stranded polynucleotide is electrophoretically driven through a nanopore that provides the only path through which ions or polynucleotides can move from the cis to the trans chamber. Residual current-vs-time signal trace from a nanopore shows clear discrimination between single bases (dGMP, dTMP, dAMP, and dCMP). (**B**) ssDNA threaded through a nanopore protein, and individual bases are identified, as the strand remains intact. Panels A and B are redrawn from images on Oxford Nanopore’s website.

The recent platform of Oxford Nanopore Technologies (ONT) delivers the real-time long-read sequencing of individual molecules [3]. It has a distinctive principle: ONT sequencing measures the disruption in ionic current when a single-stranded DNA molecule passes through a nanopore of an electrically resistant yet voltage-applied membrane. The change in ionic current is translated into sequence information in real time by base-calling software. The process of ONT sequencing adopts a PCR-free method to directly sequence genomic DNA molecules (Figure 1). Therefore, as long as the DNA strand remains intact during sample preparation, there is no upper limit to the read length. Compared with SGS, ONT has many advantages. The MinION sequencer, one of the ONT platforms, is the first handheld sequencer; it measures only 2 cm × 4 cm × 9 cm and weighs about 100 g. It can typically work in a variety of environments, even in space and polar regions. In addition, the construction process for ONT sequencing libraries is relatively brief and straightforward, requiring only 1 h. Hence, because of its portable size, low price, and low computing requirements, MinION could revolutionize the discipline of genomics. In recent years, ONT has been greatly developed, but it is still used less in veterinary microbiology research and clinical diagnosis.

Here, we report a comparative study on the genome assemblies of *Haemophilus parasuis* using different strategies from three different sequencing platforms: NovaSeq from Illumina (NGS), and long-read platforms PacBio Sequel and ONT MinION. *Haemophilus parasuis* causes Glässer’s disease, which is characterized by fibrinous polyserositis and arthritis in swine [4]. The strains are classified into 15 serovars, and nontypeable isolates represent a high percentage (about 25%). Isolates of different serovars, and even within the same serovar, are heterogeneous in their genomic traits and virulence. There is a lack of clear genetic markers for the virulence that can be used to distinguish highly pathogenic strains, which brings challenges for clinical diagnosis and vaccine development. To solve this problem, it is necessary to analyze genomic differences among a large number of virulent strains by relying on efficient and low-cost sequencing techniques. We compared the assembled genomes in terms of the accuracy, quality, and completeness of the long-read-only assembly, hybrid assembly, and assembly with polishing. Lastly, we aligned the transcripts obtained through RNA-seq with the genome assemblies to explore the effects of sequencing errors on protein prediction. 

## 2. Results

### 2.1. Data Sequencing

We sequenced the genome of the HPS_16 strain of *Haemophilus parasuis* with three sequencing platforms: Illumina Novaseq 6000, PacBio Sequel, and ONT MinION MK1B. 

#### 2.1.1. Illumina Sequencing

For Illumina data, we obtained 2 × 150 bp paired reads with a depth of about 400×. The average read-quality value was about Q29.7, and 92.3% of the reads had a quality value greater than Q30.

#### 2.1.2. PacBio Sequencing

For the PacBio SMRT sequencing data, we obtained reads with a depth of about 50×. The longest read was 80,413 bp. The average read length was 9598 bp, and the average read-quality value was about Q15.

#### 2.1.3. ONT Sequencing

For the ONT sequencing data, reads with a depth of about 50× were obtained. The longest read was 125,711 bp. The average read length was 5480 bp, the average read-quality value was about Q13.2, and 85.4% of the reads had a quality value greater than Q10. The upper limit of the read length for ONT sequencing was longer than that of PacBio, and the quality of the reads was comparable to that of the PacBio reads.

### 2.2. De Novo Assembly with Different Strategies

#### 2.2.1. Independent Assembly

**Figure 2:**
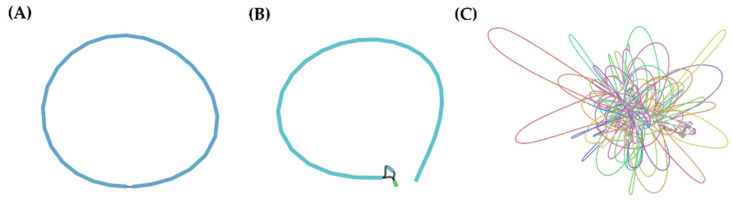
Comparison of results of independent assembly strategies. (**A**) Genome assembled with nanopore reads; (**B**) longest contig assembled with PacBio reads; (**C**) genome assembled with Illumina reads. Plots were obtained by using Bandage on the “assembly_graph.gfa” output file from SPAdes or the “contig.gfa” output file from Canu. Connections between contigs represent overlaps between contig ends.

First, we used the generated reads by the different platforms to independently perform de novo assembly. Results are shown in Table 1. Using the ONT assembly approaches, we could completely assemble the genome (all contigs were circularized) without any manual intervention, even though sequencing depth was lower than that obtained with Illumina. Although we did not obtain a continuous assembly using the PacBio reads, the number of contigs was far lower than that of the Illumina assembly, and the longest contig length reached the expectations. This result demonstrates the advantages of long-read sequencing technologies in bacterial-genome assembly (Table 1, Figure 2). 

#### 2.2.2. Hybrid Assembly

Second, we used the Illumina reads to generate a short-read assembly that was later scaffolded with long reads from ONT or PacBio. Results are shown in Table 2. The genomic continuity was improved, and the number of contigs was reduced from 527 to 266 (Illumina + ONT) or 236 (Illumina + PacBio + ONT) by using SPAdes. However, the complete genome was still not obtained. In comparison, Unicycler had the most robust hybrid assembly strategy, closely followed by MaSuRCA. Unicycler assembled complete genomes using Illumina reads in combination with ONT or PacBio reads, while MaSuRCA assembled complete genomes by only using Illumina reads combined with ONT reads. SPAdes, Unicycler, and MaSuRCA performed well in terms of genome size and GC content, as they all generated accurate genome sizes and GC contents that were similar to those of the reference genomes (*Haemophilus parasuis* SH0165, 2,269,156 bp, 39.99% G + C). 

#### 2.2.3. Long-Read Assembly with Polishing

Lastly, we used the ONT or Illumina reads for multiple rounds of polishing with Pilon (Illumina reads) and Medaka (ONT reads) based on the long-read-only assembly to improve the draft assemblies (Table 3). Medaka was used to calibrate the draft genome with ONT reads. This was performed by using neural networks that were applied to a pileup of individual sequencing reads against a draft assembly that was 50 times faster than Nanopolish. Pilon was used to calibrate the draft genome with Illumina short reads through three rounds. Categories for the polishing of Pilon include single-base differences, indels, gap filling, block substitution events, and misassemblies. Homopolish is an SVM-based polishing model for the correction of systematic ONT errors by using homologous sequences. In conjunction with Medaka, genome quality can exceed Q50 in an R9.4 flow cell, thus eliminating the need for the polishing of Illumina reads.

### 2.3. Assembly Assessment and Comparison

#### 2.3.1. QUAST Assessment of Assembly Quality

The assessment of the assemblies with different strategies was performed with QUAST, a quality-assessment tool for evaluating and comparing genome assemblies with a reference genome (*Haemophilus parasuis* SH0165, 2,269,156 bp, 39.99% G + C, 4507 genomic features). QUAST includes assessment metrics, such as contig sizes, misassemblies, and structural variations, genomic functional elements, and NA50 based on aligned blocks. Quality-control results for assemblies with different strategies are shown in Table 4. For the independent assembly strategy, the complete genome can be directly assembled with ONT reads. The Illumina assembly had the fewest misassemblies (51) but the largest number of contigs (527) and assembled fewer complete genes than ONT and PacBio did. For the hybrid assembly strategy, Unicycler performed the best, followed by MaSuRCA. However, the genomes assembled with SPAdes were highly fragmented. Moreover, hybrid assemblies of Illumina and ONT reads were easier for obtaining complete genomes, probably because ONT reads provided more adequate genome-arrangement information. For the long-read assembly with polishing strategy, the ONT and PacBio assemblies that were polished by Homopolish or Pilon had the best results in almost all metrics. They assembled a higher percentage of the genome (>89.5%) and had larger NGA50 values than those of the other assemblies. The strategy of the long-read assembly with polishing assembled the highest number of complete genes and had fewer misassemblies than the other strategies did. The ONT assembly with polishing using ONT reads had slightly higher indel numbers and fewer complete genes than the assemblies that were corrected with Illumina short reads did.

#### 2.3.2. BUSCO Assessment of Assembly Completeness

**Figure 3:**
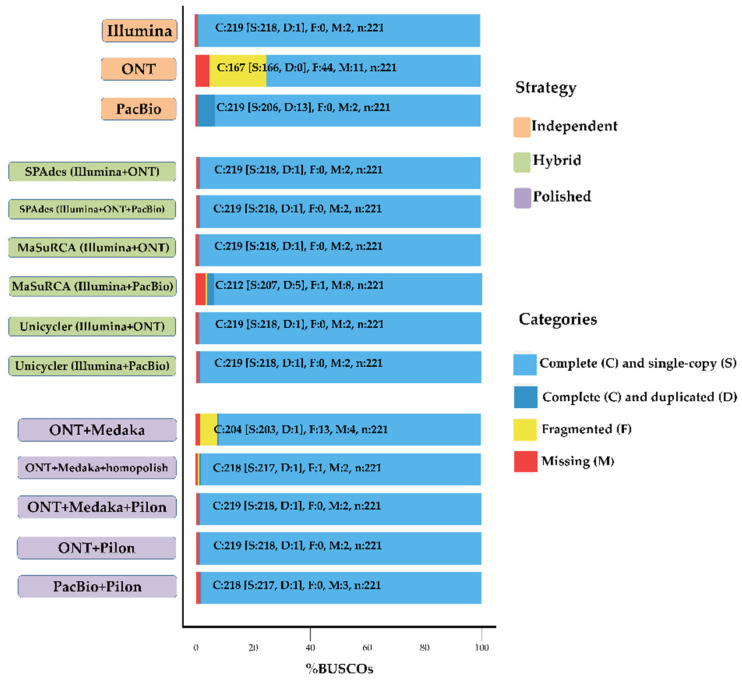
Evaluation of completeness of assembly results of different strategies. Assessments of the completeness of the assembly genomes with the datasets of proteobacteria_odb9 lineage. Bar charts produced with BUSCO plotting tool to show proportions that were classified as complete (C, blue), complete single copy (S, light blue), complete duplicated (D, dark blue), fragmented (F, yellow), and missing (M, red).

Assembly completeness was measured by using BUSCO against the proteobacteria_odb9 lineage. BUSCO identifies complete, fragmented, and missing genes. Figure 3 shows that the completeness of the raw ONT assembly was significantly improved after polishing with ONT reads. The percentage of complete genes increased from 75.1% to 92.4%. Further error correction using Illumina short reads made it possible obtain a highly complete genome (the percentage of complete genes was 99%). Another effective strategy was the use of Homopolish software for the correction of systematic ONT errors by using homologous sequences, and the percentage of complete genes increased from 92.4% to 98.6%. The advantage of this strategy is that highly accurate genomes can be directly obtained without polishing with SGS reads. However, Homopolish currently only supports error correction in microbial genomes, and its efficiency is related to the abundance of related genomes in the NCBI database. Hybrid assembly strategies also obtained a high proportion of complete genes. SPAdes and Unicycler produced more accurate assemblies compared to MaSuRCA. However, SPAdes has some obvious weaknesses that resulted in highly fragmented assemblies, whereas the MaSuRCA and Unicycler assemblies were more contiguous. 

#### 2.3.3. Alignment of 16S rDNA Sequences

**Figure 4:**
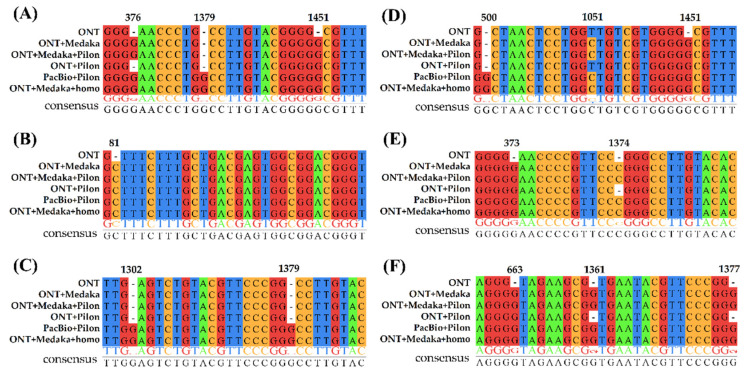
16S rDNA sequence alignment results of assembled genomes. (**A**–**F**) Alignment of 16S rDNA copies of each assembly with starting positions at about 366,200, 204,400, 2,015,700, 2,083,100, 776,000, and 1,925,000, respectively.

Furthermore, all assemblies had the same amounts of ribosomal RNA, with eight copies of 5S rRNA, six copies of 16S rRNA, and six copies of 23S rRNA. The 16S rDNA sequences at the corresponding positions of the different assemblies were extracted for multiple sequence alignments. Results are shown in Figure 4 (only mismatches and indel sites are shown). The 16S rDNA of the ONT draft assembly mainly comprised some indels and only one mismatch (Figure 4, D 1051 site). This observation implied that the primary errors in the ONT sequencing were indels, which were generated when homopolymer regions were sequenced. These sequencing errors can be filled and corrected through polishing. For the ONT assembly, the effect of multiple rounds of polishing using ONT and Illumina reads was better than that obtained by using one of them alone. Surprisingly, the sequence completeness of the 16S rDNA polished by Homopolish was even better than that of Medaka and Pilon, and all indels were filled. 

### 2.4. Aligning Transcripts with Assemblies

We used RNA-seq on the Illumina platform with cDNA libraries of the HPS-16 strain to evaluate the negative impact of long-read assembly errors on protein prediction. By aligning with the H. parasuis SH0165 reference genome and filtering by the bit score, a total of 2294 transcripts were obtained for downstream analysis.

A summary of the results for each assembly alignment is shown in Table 5. All assemblies had similar numbers of total and near-full-length mRNA alignments and total genes with mismatches. A naive comparison with the error-corrected assemblies showed that the ONT draft assembly was notably enriched in indel errors. First, the most errors occurred in the ONT-only assembly, with 664 protein-coding genes predicted to be disrupted by indels. The ONT assembly polished by its reads (ONT + Medaka) came second, with 278 affected protein-coding genes. The third was the ONT assembly polished by Medaka and Homopolish; the protein-coding genes affected by indels were reduced to 189. Fourth, the polished PacBio and ONT genome with Illumina reads (ONT + Pilon, ONT + Medaka + Pilon, PacBio + Pilon) showed better statistics, but there were still about 170 protein-coding genes with indel errors in the assembly.

### 2.5. Aligning Transcripts with Predicted CDSs

A summary of the results for CDSs of the prediction and alignment of the assemblies is shown in Table 6. All polished assemblies had similar numbers of total predicted CDSs (2332–2462) except for the ONT draft assembly (2861). These results indicated that the ONT sequencing errors adversely affected the prediction of protein-coding genes. Moreover, transcripts were aligned with the protein-coding sequences (CDSs) of the assemblies predicted by Prodigal by using Blastn (evalue = 1.00 × 10^−5^. Compared with the draft assembly, the alignment number increased by about 20% after polishing with Medaka using raw ONT reads. The more accurate ONT assembly benefitted from the Pilon correction with Illumina short reads, and the numbers of near-full-length and full-length alignments increased by about 30% and 35%, respectively. The Homopolish correction achieved accuracy similar to that of Pilon without using Illumina reads. 

### 2.6. Robustness of ONT Sequencing

To test the robustness of ONT sequencing, 15 clinical isolates of *Haemophilus parasuis* (HPS_1~HPS_15) were sequenced using ONT MinION MK1B platform. Reads of each strain with a depth of about 100× were obtained. The raw ONT reads were corrected using Canu, and SMARTdenovo was then used to assemble the error-corrected reads to obtain the assembled genome sequence. Then, we used the raw reads for multiple rounds of polishing with Medaka and Homopolish to improve the draft assemblies. 

**Figure 5:**
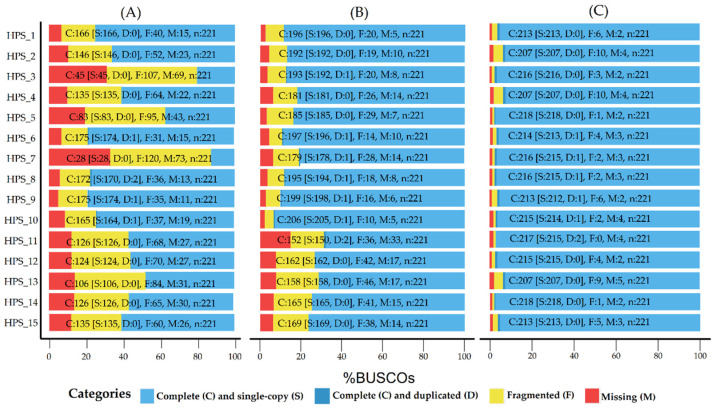
Evaluation of completeness of assembly results of 16 isolates. Assessments of the completeness of assembly genomes with datasets of the proteobacteria_odb9 lineage. Bar charts were produced with the BUSCO plotting tool to show proportions that were classified as complete (C, blue), complete single copy (S, light blue), complete duplicated (D, dark blue), fragmented (F, yellow), and missing (M, red). (**A**) Draft assemblies; (**B**) assemblies polished with Medaka; (**C**) assemblies polished with Medaka and Homopolish.

Table 7 shows a summary of the results for the polished assemblies of 15 isolates of H. parasuis. Using the ONT assembly approaches, we could completely assemble all 15 genomes without any manual intervention. The median genome size of each isolate was 2.37 M (IQR: 2.34–2.39 M), and the median GC% was 40.02% (IQR: 40.01–40.06%), consistent with the reference genome (*Haemophilus parasuis* SH0165, 2.3 M, 39.99% G + C). Moreover, Figure 5 shows that the increasing completeness of 15 genomes with multiple rounds of polishing by Medaka and Homopolish. The median percentage of the complete genes of the draft genomes was 61.10% (IQR: 54.05–75.22%; Figure 5A). This index improved to 85.3% (IQR: 76.05–88.80%; Figure 5B) with two rounds of polishing using Medaka and to 97.28% (IQR: 96.38–97.84%; Figure 5C) after further error correction with Homopolish. These results indicated that ONT sequencing is extremely robust and can readily obtain complete assemblies of bacterial genomes. When Homopolish was combined with Medaka for polishing, both mismatch and indel errors were significantly eliminated, and genomes quality was greatly improved. In addition, a CPU is sufficient for Homopolish, as it is based upon an SVM (e.g., ∼5 min for polishing a bacterial genome).

## 3. Discussion

The *H. parasuis* genome could be assembled de novo with a read depth of about 50× by using PacBio or ONT platforms for the independent assembly strategy. Moreover, the complete circular genome can be directly assembled by using ONT reads. However, a continuous genomic sequence was not obtained by using Illumina short reads alone. On the SGS platform, the continuous assembled genome could be improved by increasing the length of the inserted fragments, such as mate-pair libraries, the insert sizes of which could range from 8 to 40 kb [5]. However, compared with the ONT platform (>100 kb for prokaryotes and >1 Mb for eukaryotes), the library-construction process in the SGS platform for the use of the long-read method was complicated and costly, and the return on investment was relatively low. Long-read sequencing has transformed genome assembly. This should be the starting point for all new genome-assembly projects. Compared with PacBio, the ONT platform allows for researchers to sequence microbial genomes more quickly and at a lower cost [6,7]. It can be used in various genome-sequencing projects due to its unique technical principles, ultralong reads, and portability, although its accuracy is slightly worse than that of the PacBio platform. 

Second, for the hybrid assembly strategy, more continuous assemblies can be achieved when using long reads in conjunction with Illumina reads. This strategy initiated the hybrid assembly with high-quality Illumina short reads and filled the gaps with ONT or PacBio long reads. We assembled the complete genome with Unicycler and MaSuRCA instead of SPAdes. Moreover, hybrid assembly using Illumina reads with ONT reads was superior to using Illumina reads with PacBio reads. This may be because the ultralong reads of ONT provide more adequate information about the genome arrangement. Similarly, Zhao Chen et al. reported a genome assembly project of 12 strains with a hybrid assembly strategy (*Escherichia coli*, *Klebsiella variicola*, *Klebsiella pneumonia*, *Enterobacter cancerogenus*, *Salmonella*, *Citrobacter braakii*, *Cronobacter sakazakii*, *Listeria monocytogenes*, *Staphylococcus aureus*, *Campylobacter jejuni*, *Campylobacter coli*). The results showed that SPAdes failed to completely assemble any of the genomes. Unicycler completed the genomes of 10 of the 12 strains, and MaSuRCA produced complete assemblies of seven strains. However, SPAdes and Unicycler produced more accurate assemblies and performed better in genomic analyses of AMR, virulence potential, and pangenome compared to MaSuRCA [8]. Unicycler exhibited improved assemblies, suggesting algorithmic approaches following that model may be the most fruitful in the future.

Third, for the strategy of assembly followed by polishing, the genomes assembled with ONT or PacBio long reads and polished with Illumina short reads were optimal in terms of accuracy, continuity, and completeness. An equally excellent method was the correction with Homopolish after the ONT-only assembly, which corrected systematic ONT errors by using homologous sequences and had the advantage of avoiding hybrid sequencing with Illumina. The developers of Homopolish tested the software’s polishing ability against bacterial genomes (*Enterococcus faecalis*, *Pseudomonas aeruginosa*, *Salmonella enterica*, et al.), a viral genome (*Lambda phage*), and a fungal genome (*Saccharomyces cerevisiae*). When combined with Medaka/HELEN, genome quality can exceed Q50 on R9.4 flow cells, achieving similar precision to that of hybrid assemblies with Unicycler [9]. Results showed that ONT-only sequencing could produce sufficiently high-quality microbial genomes for downstream analysis. Hence, considering the costs and efficiencies of these experiments, we recommend the use of ONT assembly followed by Homopolish correction in microbial-genome sequencing. However, the efficiency of Homopolish is related to the abundance of related genomes in NCBI, and it has not been tested on noncoding regions, which represent a large proportion of eukaryotic genomes. Therefore, for all eukaryotes and for prokaryotes that lack related genomes in NCBI, the strategy of assembly with long-read technology combined with accurate short-read-technology error correction is more feasible. For example, Miten Jain reported the sequencing and assembly of a reference genome for the human GM12878 Utah/Ceph cell line using the MinION sequencer. The final assembled genome was 2867 million bases in size, covering 85.8% of the reference. Assembly accuracy, after incorporating complementary short-read sequencing data, exceeded 99.8% [10]. In addition, long-read sequencing is a great tool to overcome the low resolution of reconstructing the repetitive regions and polyploidy of plant genomes. In combination with the SGS technology, the genomes of various plants were completely assembled [11,12,13]. This leads to a deeper understanding of plants’ genomic diversity, evolution, and gene function, in turn accelerating the process of plant breeding and the production of improved varieties. In summary, there is no perfect sequencing technology at present, and the advantages and disadvantages of different mainstream sequencing platforms are shown in Table 8. For actual genomic-sequencing projects, we need to select the appropriate sequencing platform according to the genomic characteristics of different species, and take advantage of the benefits of different platforms, such as the ultralong reads of ONT, and the high-accuracy reads and high throughput of Illumina to solve complex genomic-sequencing challenges. Moreover, the low-cost, scalable, and easy-to-operate ONT platform offers researchers worldwide the opportunity to independently resolve the genomes of most organisms at any location, unlike the previous need to rely on third-party sequencing facilities, which will increase the efficiency of genome sequencing. At the same time, the large number of front-line users will ensure the sustainability of ONT sequencing technology.

By aligning the 16S rDNA sequences from different assemblies, most regions of the ONT-read-only assembly were highly accurate, and the predominant errors were indels that were concentrated in the homopolymer region. For the R 9.4.1 flow cells, the raw signal was mainly influenced by three central nucleotides (k-mers) that occupied the pores. By introducing frameshifts and premature stop codons, these errors could potentially critically affect the interpretation of the translated regions [14,15,16]. In order to improve signal robustness, ONT chemistry involves the attachment of a motor protein to the DNA, which slows down the translocation and allows for the k-mers to reside within the pore for long enough to differentiate the signal from noise. Nevertheless, despite the reduced translocation speed, it is difficult to detect the transition between two identical k-mers, which complicates the detection of homopolymers that are longer than the k-mer. Because the translocation speed for nucleotides is generally nonuniform, it is not accurate to infer the homopolymer length from the duration of the measured signal, leading to the generation of indels [17].

As we aligned the transcripts with assemblies, indels were present in many aligned genes (664 of 2116) in the unpolished ONT-long-read assembly genome. Correction using ONT reads was able to reduce the number of genes with indels from 664 to 278; this number was further reduced to about 180 after another round of correction using Homopolish or Pilon. Results showed that using only ONT reads for polishing could achieve the purpose of filling indels. However, further polishing is necessary in order to obtain a higher-quality genome. Then, we aligned the transcripts with the predicted CDSs of the different assemblies and obtained the same conclusions as above. Errors in long-read assemblies can critically affect protein prediction. One way to tackle this problem is to polish the genome assembly with long reads by using Medaka or with short reads by using Pilon. Another option is the use of Homopolish for polishing, as it corrects sequencing errors by retrieving homologs from closely related genomes and a trained ML model. The polished genomes were reported to achieve an accuracy of Q40–90 (>99.99%) [12]. From the perspective of the nature of the data, most of the related genomes utilized by Homopolish were sequenced with the Illumina platform in its early stages. Therefore, this is equivalent to using multiple ultralong Illumina reads to polish a genome assembly. Recently, ONT has started offering novel reagents that allow for the continuous sequencing of the forward and reverse strands of a single DNA molecule. With these reagents, mode raw-read-length accuracy of greater than 99% is expected to be generated. Other updates include ultrahigh-precision base-calling options, a new generation of sequencing instruments, and a new control method for the sequencing process. These important updates make it possible to directly generate ultrahigh-precision genomes by using ONT sequencing. 

The *H. parasuis* disease occurs in swine populations around the world, irrespective of health status. Considering the increasing pressure to reduce reliance on antibiotics, vaccination strategies for preventing systemic infection and mortality are more emphasized. However, cross-protection between different serovars and even within the same serovar is variable and difficult predict. To design effective new universal vaccines, genomic methods are needed in order to screen for antigens with protective potential. Compared with the SGS technology, long-read ONT sequencing can provide more abundant genetic information for solving this challenge. 

In conclusion, the assembly of bacterial genomes can be directly achieved by using long-read sequencing techniques, such as PacBio and ONT. Compared with PacBio, the ONT platform has the advantages of lower cost, faster sequencing speed, longer read length, and greater ease of operation. Its current accuracy is comparable to that of PacBio, but with updates to ONT sequencing, its accuracy could reach or even exceed that of SGS platforms. Moreover, in current research, to maximize assembly accuracy, it is essential to polish the assembly with homologous sequences of related genomes or sequencing data from short-read technology. When necessary, indels and errors can be checked by aligning known proteins and cDNA or mRNA sequences against the genome and fixing them manually. Furthermore, we found discrepancies between the published reference genome and our assemblies. This suggests that the genomes of different isolates are highly variable and that the genomes of individuals are not representative of the species as a whole. Therefore, for the foreseeable future, to better explain the process of genetic evolution and variation in bacteria, the large-scale genome sequencing and construction of pangenomes for specific species of bacteria are needed. At the same time, no sequencing-assembly pipelines should be set in stone, but they need to be continuously updated and optimized for the organism to be sequenced.

## 4. Materials and Methods

### 4.1. Bacterial Isolates and DNA Extraction

For sequencing, we selected and subcultured an isolate of *Haemophilus parasuis*, strain HPS_16. The subcultures were aerobically undertaken on a TSA medium containing 5% bovine serum and NAD at 37 °C for 24–36 h. DNA was extracted from the subcultured isolates using the E.Z.N.A Bacterial DNA Kit (OMEGA). Quality was assessed using the Qubit fluorometer 3.0 (Thermo Fisher Scientific, Waltham, MA, USA) and NANODROP 2000 spectrophotometer (Thermo Fisher Scientific, Waltham, MA, USA).

### 4.2. Library Preparation and Sequencing

#### 4.2.1. ONT Library Preparation and Sequencing

The ONT MinION sequencing library was prepared as follows: 1 ug of genomic DNA was used to prepare the library according to the SQK-LSK109 Sequencing Kit protocol, including the NEBNext FFPE DNA repair (New England Biolabs, Ipswich, MA, USA), Ultra II End-prep (NEB), and adapter ligation steps. Library fragments were purified with AMPure XP beads (Beckman Coulter, Brea, CA, USA), followed by clean-up. After calculation, 5–50 fmol of the final prepared library was loaded onto flow cells of version FLO-MIN106 R9.4.1 SpotON on a MinION MK1B device and sequenced for 4 h.

#### 4.2.2. PacBio Library Preparation and Sequencing

The PacBio 10 KB SmrtBell sequencing library was prepared as follows. The DNA sample was broken into target fragments of the size required for library construction. The genomic DNA was repaired with a damage-repair reagent and end-repaired using repair enzymes. After the DNA damage repair, the ends of the double-stranded fragments were polished and subsequently tailed with an A-overhang. Ligation with T-overhang SMRTbell adapters was performed at 20 °C for 60 min. Following ligation, the SMRTbell library was purified with two AMPure PB bead clean-up steps. Lastly, the prepared library was sequenced on the PacBio Sequel platform at Novogene Bioinformatics Technology, BJ, CHN. 

#### 4.2.3. Illumina Library Preparation and Sequencing

The Illumina 350 bp small-fragment library was prepared as follows. The DNA sample was randomly interrupted into fragments with a length of about 350 bp, and the library was then prepared through terminal repair, adapter ligation, purification, and PCR amplification. Lastly, the prepared library was double-end sequenced on the Illumina Novaseq 6000 platform at Novogene Bioinformatics Technology, BJ, CHN.

### 4.3. Read Preparation and Assembly

ONT fast5 read files were base-called with Guppy (v4.0.11). Adapter sequences were trimmed with Porechop (v0.2.4 https://github.com/rrwick/Porechop (accessed on 15 July 2020)). Read quality was calculated with NanoPlot (v1.3.0 https://github.com/wdecoster/NanoPlot (accessed on 15 July 2020)). In addition, the PacBio and Illumina reads were prepared after quality control. We used the following strategies to assemble the genome of the isolate. 

#### 4.3.1. Independent Assembly of ONT/PacBio/Illumina Reads 

PacBio reads were assembled using Canu (v1.5 https://github.com/marbl/canu (accessed on 15 October 2020)) to obtain the assembled genome sequence [18]. Illumina reads were assembled using SPAdes (v3.15.2 https://github.com/ablab/spades (accessed on 15 March 2021)) to obtain the assembled genome sequence [19]. Raw ONT reads were corrected using Canu, and SMARTdenovo (https://github.com/ruanjue/smartdenovo (accessed on 15 October 2020)) was then used to assemble the error-corrected reads to obtain the assembled genome sequence.

#### 4.3.2. Hybrid Assembly

Hybrid assembly for the ONT and Illumina reads (-1 Illumina_forward_pair-end_reads.fastq -2 Illumina_reverse_pair-end_reads.fastq—nanopore ONT.fastq), as well as for the ONT, PacBio, and Illumina reads (-1 Illumina_forward_pair-end_reads.fastq -2 Illumina_reverse_pair-end_reads.fastq—nanopore ONT_reads.fastq—pacbio PacBio_reads.fastq), was performed using the SPAdes assembler with the default options [19]. Similarly, the reads sets were assembled using Unicycler (v0.4.0, https://github.com/rrwick/Unicycler (accessed on 20 June 2021)) [20] and MaSuRCA (v4.0.3, https://github.com/alekseyzimin/masurca (accessed on 20 June 2021)) [21].

#### 4.3.3. Assembly Followed by Polishing

The PacBio assembly genome was polished using Illumina reads with Pilon (v1.23 https://github.com/broadinstitute/pilon (accessed on 15 October 2020)) [22]. The ONT assembly genome was polished using Illumina reads with Pilon and ONT reads with Medaka (v1.0.1 https://github.com/nanoporetech/medaka (accessed on 15 October 2020)). Then, Homopolish (v0.2.1 https://github.com/ythuang0522/homopolish (accessed on 15 May 2020)) [9] was used to remove systematic indel errors from the genome polished by Medaka.

### 4.4. Assembly Assessment and Comparison

We used Bandage (v0.8.1 https://github.com/rrwick/Bandage (accessed on 20 October 2020)) [23] to visualize the assemblies and compared the assemblies from different strategies with the reference genome (*H. parasuis* SH0165, NCBI Reference Sequence: NC_011852.1) [24] using QUAST (v5.0.2 https://github.com/ablab/quast (accessed on 1 July 2021)) [25]. QUAST evaluates the metrics in several groups, including on the basis of contig sizes, misassemblies, structural variations, genome representation, and functional elements. Then, assembly completeness was measured using BUSCO (v3.0.2 http://busco.ezlab.org (accessed on 1 July 2021)) against the proteobacteria_odb9 lineage [26]. Ribosomal RNA sequences were predicted using RNAmmer (http://www.cbs.dtu.dk/services/RNAmmer (accessed on 20 October 2020)) [27], and the 16s rDNA sequences were extracted for multiple-sequence alignment using MEGA X software (https://www.megasoftware.net/ (accessed on 20 October 2020)) [28].

### 4.5. Alignments of Transcripts with Assemblies

#### 4.5.1. Illumina Library Preparation and RNA-Seq

We extracted the total RNA of the HPS-16 isolate using the E.Z.N.A Bacterial RNA Kit (OMEGA) and constructed a cDNA library with the isolated RNA. Lastly, the prepared library was double-end sequenced on the Illumina Hiseq 4000 platform at Novogene Bioinformatics Technology Co., Ltd.

#### 4.5.2. Transcript Preparation

The raw data of the RNA-seq were filtered by utilizing FASTQC software. The filtered reads were aligned and mapped to the *H*. *parasuis* SH0165 reference genome using Bowtie2 (v2.4.4 https://github.com/BenLangmead/bowtie2 (accessed on 1 November 2020)) [29] for splicing transcripts. Then, the obtained transcripts were aligned to the NCBI nr database using blastx (evalue =1.00E-05) to filter protein-coding mRNAs for downstream analysis. 

#### 4.5.3. Aligning Transcripts with Assemblies 

First, we aligned the transcripts with genomes assembled with the different strategies using BLAT and recorded the maximal match alignments by filtering the alignment results. Then, alignment number, the number of near-full-length alignments (more than 95% identity), and the frequencies of mismatches and indels were counted according to the filtering results. Second, transcripts were aligned with the protein-coding sequences (CDSs) of the assemblies, which were predicted by Prodigal (v2.50 https://github.com/hyattpd/Prodigal (accessed on 15 November 2020)) using blastn (evalue =1.00 × 10^−5^) [30]. We filtered the alignment results according to the bit score value, and sequences aligned with different segments of the same transcript were combined. Then, the number of full-length alignments and the number of near-full-length alignments were counted according to the filtering results.

### 4.6. Robustness Testing for ONT Sequencing

To test the robustness of ONT sequencing, we selected and subcultured 15 isolates of *Haemophilus parasuis*, strain HPS_1~HPS_15. DNA extraction of the subcultured isolates was carried out according to Section 4.1. Then, quality-controlled DNA samples were used for the construction of the ONT library and were sequenced on a MinION MK1B device. Raw ONT reads were corrected using Canu, and SMARTdenovo was then used to assemble the error-corrected reads to obtain the assembled genome sequence. Lastly, Medaka and Homopolish were used to perform multiple rounds of polishing for draft assemblies.

## Figures and Tables

**Table 1:** Statistics of genome-assembly results of independent assembly strategies.

Platforms	Assembler	Contigs	Largest Contig (bp)	N50	GC%
Illumina	SPAdes	527	157,573	40,498	39.87
PacBio	Canu	25	2,351,556	2,351,556	40.01
ONT	Canu	1	2,360,091	2,360,091	40.02

**Table 2:** Statistics of genome-assembly results of hybrid assembly strategies.

Platforms	Assembler	Contigs	Total Length (bp)	N50	GC%
Illumina + ONT	SPAdes	266	2,402,219	1,953,224	39.97
Illumina + PacBio + ONT	SPAdes	236	2,410,042	2,351,543	40.02
Illumina + ONT	Unicycler	1	2,349,186	2,349,186	40.03
Illumina + PacBio	Unicycler	1	2,349,340	2,349,340	40.03
Illumina + ONT	MaSuRCA	1	2,365,339	2,365,339	40.02
Illumina + PacBio	MaSuRCA	4	2,395,409	1,345,876	40.04

**Table 3:** Statistics of results of genome assembly with polishing.

Platforms	Polishing	Contigs	Length (bp)	GC%
PacBio	Pilon × 3	1	2,349,014	40.03
ONT	Pilon × 3	1	2,361,312	40.04
ONT	Medaka	1	2,361,313	40.03
ONT	Medaka + Homopolish	1	2,361,359	40.04
ONT	Medaka + Pilon × 3	1	2,361,444	40.04

**Table 4:** Quality control results obtained with QUAST for the assemblies using different strategies.

Strategy	Platform	Polishing/Hybrid Method	Genome Fraction	Genomic Features	Aligned Length	NGA50	Misassemblies	Mismatches	Contigs	Largest Contig	N50	GC (%)	Predicted rRNA
Independent	Illumina	N/A	87.07	3741 + 224 part	1,981,064	23,234	51	30,355	527	157,573	40,498	39.87	2 + 0 part
	PacBio	N/A	89.583	3862 + 205 part	2,377,501	43,084	200	36,379	25	2,391,556	2,391,556	40.1	11 + 0 part
	ONT	N/A	89.458	3862 + 204 part	2,084,692	43,068	158	31,999	1	2,360,091	2,360,091	40.02	8 + 0 part
Hybrid	Illumina + ONT	SPAdes	89.276	3855 + 205 part	2,071,505	35,530	163	31,833	262	1,953,224	1,953,224	40.01	8 + 0 part
	Illumina + ONT + PacBio	SPAdes	89.426	3866 + 201 part	2,076,717	35,559	167	31,943	236	2,351,543	2,351,543	40.02	8 + 0 part
	Illumina + ONT	Unicycler	89.553	3862 + 206 part	2,076,870	37,563	159	31,797	1	2,349,186	2,349,186	40.03	8 + 0 part
	Illumina + PacBio	Unicycler	89.61	3864 + 204 part	2,077,902	37,563	157	31,811	1	2,349,340	2,349,340	40.03	8 + 0 part
	Illumina + ONT	MaSuRCA	89.584	3860 + 204 part	2,089,749	37,227	157	31,883	1	2,365,339	2,365,339	40.02	8 + 0 part
	Illumina + PacBio	MaSuRCA	87.089	3742 + 215 part	2,094,785	35,418	169	32,512	4	1,345,876	1,345,876	40.04	7 + 0 part
Polished	PacBio	Pilon	89.578	3862 + 204 part	2,077,525	43,084	156	31,830	1	2,349,014	2,349,014	40.03	8 + 0 part
	ONT	Medaka	89.524	3860 + 204 part	2,087,009	43,079	157	32,033	1	2,361,313	2,361,313	40.03	8 + 0 part
	ONT	Medaka+Homopolish	89.61	3864 + 204 part	2,087,858	43,086	158	32,085	1	2,361,359	2,361,359	40.04	8 + 0 part
	ONT	Pilon	89.598	3864 + 204 part	2,087,224	43,084	157	32,130	1	2,361,312	2,361,312	40.04	8 + 0 part
	ONT	Medaka + Pilon	89.524	3860 + 204 part	2,087,133	43,084	156	32,045	1	2,361,444	2,361,444	40.04	8 + 0 part

Note: The complete H. parasuis SH0165 genome sequence was selected as the reference genome, with a GC content of 39.99%, 4507 genomic features, and a total of 2,269,156 bp. Cell colors go from red to blue to indicate how metrics are ranked from worst to best.

**Table 5:** Results of BLAT alignment between transcripts and assembled genomes.

	ONT	ONT + Medaka	ONT + Medaka + Homopolish	ONT+ Pilon	ONT + Medaka + Pilon	PacBio + Pilon
Total aligned number	2116	2116	2117	2117	2117	2117
Near full length (>95%)	1910	1949	1917	1916	1918	1918
Number of total genes with mismatches	1837	1834	1834	1833	1831	1833
Number of total genes with indels	664	278	189	178	173	172

**Table 6:** Blast alignment of transcripts with CDSs of the assembled genome.

Assembly Name	Predicted CDSs	Near-Full-Length Alignment (>90%)	Full-Length Alignment
ONT	2861	1464	1285
ONT + Medaka	2462	1777	1603
ONT + Medaka + Homopolish	2359	1865	1713
ONT + Pilon	2358	1891	1738
ONT + Medaka + Pilon	2348	1892	1740
PacBio + Pilon	2332	1892	1740

**Table 7:** Summary of assemblies of 15 isolates of *H. parasuis*.

Isolate Name	Polishing Method	Contigs	Length (bp)	GC (%)
HPS_1	Medaka + Homopolish	1	2,349,963	40.03
HPS_2	Medaka + Homopolish	1	2,349,429	40.05
HPS_3	Medaka + Homopolish	1	2,373,571	40.02
HPS_4	Medaka + Homopolish	1	2,463,730	40.08
HPS_5	Medaka + Homopolish	1	2,366,591	40.06
HPS_6	Medaka + Homopolish	1	2,387,172	40.02
HPS_7	Medaka + Homopolish	1	2,298,620	39.94
HPS_8	Medaka + Homopolish	1	2,373,150	40.01
HPS_9	Medaka + Homopolish	1	2,379,165	40.03
HPS_10	Medaka + Homopolish	1	2,283,294	39.97
HPS_11	Medaka + Homopolish	1	2,423,838	40.13
HPS_12	Medaka + Homopolish	1	2,306,955	40
HPS_13	Medaka + Homopolish	1	2,503,741	40.12
HPS_14	Medaka + Homopolish	1	2,410,234	40.11
HPS_15	Medaka + Homopolish	1	2,316,424	39.95

**Table 8:** Comparison of different sequencing platforms.

	Oxford Nanopore	PacBio	Illumina
Method of sequencing	Real-time, single-molecule DNA, nanopore exonuclease sequencing	Single-molecule DNA sequencing	Reversible terminator sequencing by synthesis
Method of detection	Current	Fluorescence/optical	Fluorescence/optical
Based on PCR	No	Yes	Yes
Approx. read length (bases)	>100 kb	10–14 kb	200–300
Average read-quality value	≈Q13	≈Q15	≈Q30
Sequencing speed	Fast	Slow	Slow
Price of sequencers	Low	High	High
Environmental requirements of sequencers	Suitable for a variety of environmental conditions, even extreme environments	High environmental requirements	High environmental requirements
Scalability of sequencers	Portable sequencer	Confined to the laboratory	Confined to the laboratory
Library preparation process	Simple and quick	Complicated	Complicated
Direct RNA sequencing	Yes	No	No

## Data Availability

The data presented in this study are available on request from the corresponding author.
